# Stress factors in veterinary medicine—a cross-sectional study among veterinary students and practicing vets in Austria

**DOI:** 10.3389/fvets.2024.1389042

**Published:** 2024-05-30

**Authors:** Viktoria Neubauer, Afsaneh Gächter, Thomas Probst, Deianira Brühl, Rachel Dale, Christoph Pieh, Elke Humer

**Affiliations:** ^1^FFoQSI GmbH—Austrian Competence Centre for Feed and Food Quality, Safety and Innovation, Tulln, Austria; ^2^Centre for Food Science and Veterinary Public Health, Clinical Department for Farm Animals and Food System Science, University of Veterinary Medicine Vienna, Vienna, Austria; ^3^Department for Psychosomatic Medicine and Psychotherapy, University for Continuing Education Krems, Krems, Austria; ^4^Division of Psychotherapy, Department of Psychology, Paris Lodron University Salzburg, Salzburg, Austria; ^5^Faculty of Psychotherapy Science, Sigmund Freud University Vienna, Vienna, Austria

**Keywords:** veterinarians, stressor, mental health, financial concerns, veterinary students

## Abstract

**Background:**

Although the issue of high mental health burden among veterinarians is well-documented in previous studies, little is known about the specific occupational stress factors associated with mental health issues. Therefore, the aims of this study were twofold: (1) to assess occupational stress factors within the veterinary profession, with a particular emphasis on comparing the expectations of veterinary students with the experiences of practicing veterinarians and (2) to link the experienced stress with mental health indicators in veterinarians.

**Methods:**

All registered veterinarians and veterinary-medicine students in Austria were invited to participate in a cross-sectional online survey. The data collection took place during the winter of 2022/2023 and included standardized questionnaires on mental well-being (WHO-5), depression (PHQ-9), anxiety (GAD-7), stress (PSS-4), and insomnia (ISI-2). Additionally, participants were asked about various estimated (students) or experienced (vets) occupational stress factors, which were to be rated on a 5-point Likert scale ranging from “not at all” to “very strongly”. An open question invited respondents to identify in free text further experienced/anticipated sources of work-related stressors in veterinary practice.

**Results:**

A total of 430 students and 440 veterinarians participated in the study. The results of a repeated measures analysis of variance (ANOVA) indicate that the burden of bureaucracy is perceived as less stressful by students than experienced by veterinarians, all other areas are perceived as more stressful by students than by veterinarians. In veterinarians, bureaucracy is experienced as the most burdensome, followed by animal suffering, and communication with animal owners. Further analysis of possible associations between the extent of perceived stressors and indicators of mental health shows that while bureaucracy is the most burdensome, it has the smallest correlation with mental health indicators. On the other hand, financial concerns, which are not ranked among the main stressors, have the strongest correlation with impaired mental health.

**Conclusion:**

The results suggest that financial security for veterinarians is crucial to safeguard their mental health. The training of veterinary medicine students and practicing veterinarians in the areas of administration, time management, handling animal suffering, and communication with animal owners might be beneficial in reducing their job-related stressors.

## 1 Introduction

Veterinary medicine encompasses diverse responsibilities, including medical care for companion animals, ensuring the health and well-being of production animals, and contributing significantly to public health (1, 2). Veterinarians work in diverse settings, from clients’ homes, farms, or zoos/aquaria to laboratories, small practices to large clinics, public authorities and slaughterhouses (3). At the heart of their profession lies the care and welfare of various animal species, with veterinarians serving as primary healthcare providers for non-human patients (2) and playing a pivotal role in preventing and controlling zoonotic diseases and ensuring food safety (4). This multifaceted profession demands a continual pursuit of clinical knowledge and skills, underpinned by a strong ethical framework that guides human-animal interactions, emphasizing respect and accommodation for the abilities, interests, and economic circumstances of animal owners. However, this profession faces challenges, including occupational stressors that impact the mental health (5). Recognizing and addressing mental health within the veterinary community is crucial due to the demanding nature of their responsibilities.

The issue of high mental illness burden among veterinarians is well-documented in previous studies (5–7). A recently published study from Austria reported that both male and female veterinarians are the only highly educated professional group with a higher suicide rate than the general population (8). Our companion studies show that Austrian veterinary students and veterinarians experience worse mental health than the Austrian general population (9, 10). General risk factors identified in both samples were female gender, (desired) specification in small animal medicine, physical inactivity, and high smartphone usage. In the practitioners, mental illness symptoms were associated with younger age, higher working hours and fewer years in the profession, and the perceived stresses of euthanasia and perceived stress of working overtime were associated with higher suicidality (11). However, little is known about the specific occupational stress factors associated with mental health issues. Therefore, the present study expands upon the previous investigations by shedding light on the occupational stressors that are specific to the veterinary profession.

Existing research has focused on various stressors encountered by veterinarians, ranging from work-overload and client interactions to management responsibilities. These stressors have been studied in different regions, such as Belgium, Germany, the United Kingdom, the United States, and New Zealand (5, 12–17). Studies consistently reveal the high levels of stress experienced by veterinarians in their profession. Research has highlighted the prevalence of stressors such as workload, client interactions, ethical dilemmas, administrative tasks, financial worries, gender dynamics, and the physical and emotional impacts of the job (5, 12, 15, 17–19). More specifically, the studies have observed frequent overtime, weekend shifts, and work overload as major stressors in the profession (5, 12–16). The challenges associated with dealing with clients, encompassing communication difficulties, managing client expectations, and navigating difficult interactions, emerge as a persistent stressor across diverse regions (5, 12, 14, 15, 18, 19). Ethical dilemmas, such as euthanasia decisions, animal suffering, and conflicts between professional responsibilities and client preferences, present significant stressors, specific for the veterinary profession (15). Research has highlighted the emotional distress and moral challenges associated with ethical decision-making in veterinary practice (18, 20, 21). Administrative tasks, including paperwork, regulatory compliance, and financial management, also features prominently as stressors for veterinarians (12, 15). Investigations conducted in Belgium underscore the taxing nature of administrative formalities within the veterinary practice (14, 15). Financial worries, such as low compensation, debt burden, and income instability, further exacerbate stress levels among veterinarians. Research conducted in the US and other countries underscores the significant impact of financial worries on veterinarians` psychological well-being (18, 19). Moreover, gender-related issues, including perceived biases, disparities, and stereotypes, impact the experiences of male and female veterinarians. Studies elucidate gender differences in client interactions, career advancement, and work-life balance within the veterinary profession (22). For instance, a study examining factors influencing attrition from the veterinary profession in the UK found that female veterinarians were more predisposed to leave the field (17).

Research on veterinary students’ view of work-related stressors is scarce. A study conducted in the UK observed differences in concerns about the work of a veterinarian in students compared to practitioners (23). Among students, the top five concerns included fears of making mistakes, achieving work-life balance, being responsible for clinical decisions, remembering information and grappling with self-confidence issues. Conversely, veterinary professionals highlighted work-like balance, compensation, and benefits, managing on-call duties, professional development opportunities, and regulation as key issues.

While these findings collectively illustrate the diverse array of challenges faced by veterinarians worldwide, there is a need to comprehensively examine the extent to which these stressors are perceived and the impact they have on specific mental health indicators. There is still limited research addressing the development and evaluation of targeted interventions aimed at equipping veterinary students with coping skills to navigate their future careers effectively.

This paper aims to contribute to the existing body of knowledge by exploring the anticipated and perceived stressors in the veterinary profession and their associations with mental health indicators. Additionally, it seeks to identify opportunities for the development of tailored interventions to support veterinary students and professionals in managing the unique stressors of their profession.

The following research questions are addressed:

1: To what extent do veterinary medicine students expect, and how do veterinarians actually experience, work-related stress factors within the veterinary profession?

1a: What are the differences in anticipated or experienced work-related stress factors between veterinary medicine students and practicing veterinarians?

1b: How do gender differences influence the perception of veterinary work-related stress factors?

2: What is the relationship between the work-related stress factors and indicators of mental health in veterinarians?

## 2 Materials and methods

### 2.1 Study design

Two cross-sectional online surveys targeting Austrian veterinary students and licensed Austrian veterinarians were conducted between November 16, 2022, and January 31, 2023. Recruitment and results on mental health indicators in comparison to the general Austrian population have been reported in detail in our companion studies (9, 10). In brief, all students enrolled in the diploma study of veterinary medicine in Austria (*N* = 1,477) were invited to participate in the survey by the Union of Students of the University of Veterinary Medicine Vienna and the registrar’s office of the university. Furthermore, invitations to participate in the study were sent via email to all registered veterinarians in the Austrian Chamber of Veterinarians list who had provided valid email addresses (*N* = 4,534 veterinarians). The online surveys were conducted utilizing the LimeSurvey platform (LimeSurvey GmbH, Hamburg, Germany). Participation was entirely voluntary, and no incentives were offered to encourage participation.

### 2.2 Ethical considerations

This study adhered to the ethical principles outlined in the Declaration of Helsinki and received approval from the Ethics Committee of the University for Continuing Education Krems, Austria (Ethical number: EK GZ 25/2021–2024). All participants provided electronic informed consent to partake in the study before completing the questionnaires.

### 2.3 Measures

#### 2.3.1 Professional characteristics

Veterinarians were queried regarding the animal species with which they engage professionally, encompassing ruminants, pigs, horses, poultry, pets, and exotic animals. Additionally, data regarding their employment status (employed or self-employed) and professional field (curative practice, university/research, consulting, abattoir, animal, and meat inspection, official veterinarian) were collected.

#### 2.3.2 Work-related stressors

Practicing veterinarians were asked about their experienced stressors, whereas students were asked about their anticipated stress factors in their future occupation.

A series of 11 original items specifically focusing on potential sources of stress in the veterinary profession were provided. These included: communication with animal owners, communication with colleagues, communication with superiors, night/weekend shifts, working overtime, euthanasia, animal suffering, bureaucracy, professional overload, financial concerns, public pressure via social media.

Survey participants were asked to rate each item on a 5-point Likert scale (ranging from 0 to 4, with options from “not at all” to “very strongly”) to express the extent to which each item contributed to the stress they encountered (vets) or anticipated to encounter in their future career (students). The option “not applicable” was also provided in the veterinarians’ survey, which was coded “0” for further statistical analysis.

An open question was posed, inviting respondents to freely articulate additional estimated (students) or experienced (vets) stressors in the veterinary profession. For veterinarians, the question posed was: “Are there other aspects that you find stressful in your work as a veterinarian? If so, please describe them.” Meanwhile, for veterinary students, the open-ended question took the form of: “Are there other aspects of working as a veterinarian that you consider stressful? If yes, please describe which ones?”

These items were developed collaboratively by a group of four individuals drawing from personal experiences in both academic study and practice, as well as through the identification of potential stressors documented in the veterinary literature. These questions were then subjected to pre-testing, where feedback from six veterinary students and seven practicing veterinarians were gathered. The feedback received from both veterinarians and students was largely positive, with most comments focusing on design and formatting issue, one participant observed that not all of the 11 listed stressors may apply universally to practicing veterinarians—for example, those working in the pharmaceutical industry do not have direct communication with animal owners. Consequently, the survey for veterinarians was amended after the pre-testing phase to include the option “not applicable” for such cases.

#### 2.3.3 Well-being (Who-5)

The World Health Organization Well-Being Index (WHO-5) was used as a measure of well-being (24). The WHO-5 comprises five items that positively assess aspects of well-being on a six-point scale from 0 (none of the time) to 5 (all of the time), with higher scores indicating higher well-being. Cronbach’s alpha was *α* = 0.85 in the veterinarian sample and *α* = 0.84 in the students` sample.

#### 2.3.4 Perceived stress (PSS-4)

Perceived stress levels were assessed using the Perceived Stress Scale (PSS-4), which consists of four self-report items. Respondents used a five-point Likert scale, ranging from 0 (never) to 4 (very often), to rate their stress levels (25). Notably, items 2 and 3 were reverse-coded. Total PSS-4 scores ranged from 0 to 16, with higher scores indicating greater perceived stress. Internal consistency, measured by Cronbach’s alpha, was *α* = 0.83 in the veterinarian sample and *α* = 0.82 in the students’ sample.

#### 2.3.5 Depressive symptoms (PHQ-9)

The Patient Health Questionnaire’s depression module (PHQ-9) was used to assess depressive symptoms (26). The PHQ-9 comprises nine self-rating items that evaluate symptoms of depression experienced over the past 2 weeks. Respondents rated these items on a four-point scale, ranging from 0 (not at all) to 3 (nearly every day), resulting in a total score ranging from 0 to 27 (27). The internal consistency (Cronbach’s alpha) was *α* = 0.86 in veterinarians and *α* = 0.84 in the students’ sample.

#### 2.3.6 Anxiety (GAD-7)

Anxiety symptoms were assessed using the Generalized Anxiety Disorder 7 scale (GAD-7), which includes seven self-rating items (28). Respondents reported symptoms of generalized anxiety over the past 2 weeks on a four-point scale from 0 (not at all) to 3 (nearly every day). Total scores ranged from 0 to 21, with higher scores indicating more severe anxiety symptoms (29). The internal consistency, Cronbach’s alpha, was *α* = 0.86 in the veterinarians’ and *α* = 0.88 in the students’ sample.

#### 2.3.7 Insomnia (ISI-2)

The assessment of sleep quality utilized the two-item version of the Insomnia Severity Index (ISI) (30). The ISI-2 self-rating items gauged an individual’s satisfaction or dissatisfaction with their current sleep patterns and the extent to which these patterns interfered with daily functioning. Respondents used a five-point Likert scale, ranging from 0 to 4, for rating these items. The total ISI-2 score could range from 0 to 8, with higher scores suggesting stronger impairment in sleep quality (31). Cronbach’s alpha was *α* = 0.71 in the veterinarian sample and *α* = 0.43 in the students’ sample.

### 2.4 Statistical analyses

All statistical tests were performed in SPSS Statistics version 26.0 (IBM Corp., Armonk, NY, United States).

To evaluate potential differences in the experienced/expected work-related stressors regarding area (11 assessed stressors), group (students, veterinarians), and gender (female, male), a repeated measures analysis of variance (RM-ANOVA) was conducted. In this RM-ANOVA the estimated (students) or the experienced (veterinarians) work-related stress was the dependent variable. There was one within-subject factor, i.e., “stressor” (11 levels: communication with animal owners, communication with colleagues, communication with superiors, night/weekend shifts, working overtime, euthanasia, animal suffering, bureaucracy, professional overload, financial concerns, public pressure via social media). There were two between-subject factors, the first was “group” (two levels: veterinary students, veterinarians) and the second was “gender” (two levels: men, women). The Greenhouse–Geisser corrected values are reported. Significant main and interaction effects were followed up by Bonferroni-corrected simple effects two-tailed tests.

To reveal potential associations of occupational factors with work-related stressors *t*-tests for independent samples were conducted. Occupational factors including employment status (self-employed vs. employed), professional field (curative practice vs. other fields), and animal species (working with specific species vs. not) were dichotomized for the statistical analysis. All tests were two-tailed, and the significance value was set to *p* < 0.0045 (*p* < 0.05/11 *t*-tests per occupational factor).

To analyze potential associations of a given work-related stressor with the surveyed mental health indicators (WHO-5, PSS-4, PHQ-9, GAD-7, ISI-2) in veterinarians, Pearson correlation coefficients (*r*) were calculated. Correlation analyses were conducted two-tailed and Bonferroni-corrected results were reported with *p* < 0.00091 (*p* < 0.05/55 bivariate correlation analyses). For completeness, data from students were also examined. In this analysis, current mental health indicators were correlated with the anticipation of future stressors in the veterinary profession.

### 2.5 Qualitative analyses

Responses to the open-ended questions underwent a thorough qualitative content analysis process (32). The initial step involved reading all the data to establish familiarity with the material and obtain a comprehensive overview of the responses. Each response was then meticulously examined, word by word, through multiple iterations. During this phase, categories for the open-ended questions were developed through an inductive approach, and comprehensive definitions for each category, along with coding guidelines and illustrative quotations, were documented in a codebook. Subsequently, subcategories sharing similar content were amalgamated into broader, more conceptually focused main categories. In the following phase, the dataset was systematically coded using the ATLAS.ti software (33). Given that respondents had the freedom to address multiple aspects within each question, assigning more than one category to a single response was a possibility. Once the entire dataset had been coded, all quotations linked to specific categories underwent a reevaluation to rectify any coding inaccuracies. Any identified coding errors were rectified, and, if necessary, category definitions and coding guidelines were refined.

## 3 Results

### 3.1 Sample description

A total of 430 students (29.1% of the total population of Austrian veterinary medicine students) and 440 veterinarians (9.7% of the total population of Austrian veterinarians) participated in the study. The student sample comprised 85.8% women, 13.5% men and 0.7% gender-diverse persons. Participants were on average 23.14 ± 3.69 years old. The veterinarian sample comprised 72.0% women and 28.0% men with an average age of 44.53 ± 11.25 years. Due to the low number of gender-diverse individuals (*n* = 3 in the students’ sample, *n* = 0 in the veterinarian sample), they were excluded from further statistical analyses.

### 3.2 Extent of expected/perceived stress factors in the veterinary profession

Results of the RM-ANOVA revealed a significant main effect of group (*p* < 0.001), gender (*p* < 0.001) and stressor (*p* < 0.001). Also, the two-way interaction between group and stressor (*p* < 0.001) as well as gender and stressor (*p* < 0.001) reached significance, whereas neither the two-way interaction between group and gender (*p* = 0.662), nor the three-way interaction between group, gender, and stressor (*p* = 0.053) were significant.

The main effect of group revealed that students perceived the work-related stressors to be more burdensome (M = 1.95, SEM = 0.047) than veterinarians (M = 1.52, SEM = 0.036). Women indicated work-related stressors to be more burdensome (M = 1.90, SEM = 0.026) than men (M = 1.57, SEM = 0.053). The main effect of stressor is illustrated in detail in Supplementary Figure S1. In brief, animal suffering, bureaucracy, communication with animal owners, working overtime and night/weekend shifts were rated as the most burdensome stressors, not differing from each other. Financial concerns were at an intermediate position, followed by professional overload, euthanasia, and public pressure via social media. Communication with superiors and colleagues were rated to be the least burdensome.

**Figure 1:**
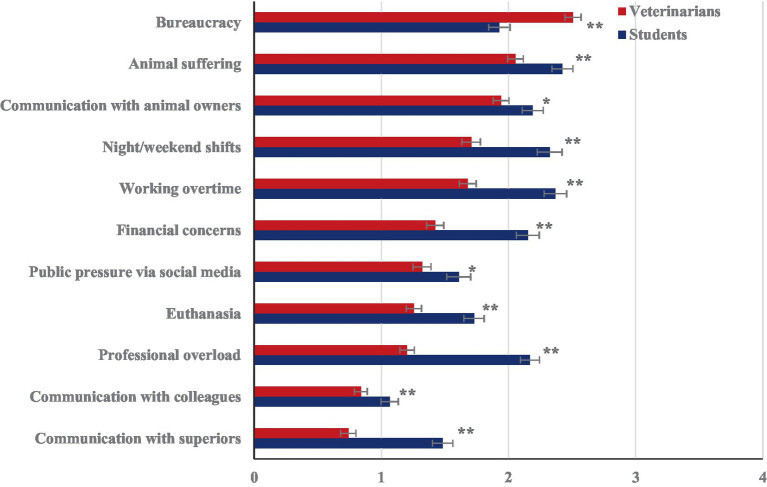
Estimated veterinary work-related stressors in veterinary students versus experienced work-related stressors in veterinarians rated on a 5-point scale from 0 “not at all or not applicable” to 4 “very strongly”. The stressors are listed in descending order, as reported by the veterinarians.

The interaction of group and stressors (Figure 1) revealed that students estimated almost all the stressors to be higher than actually experienced by veterinarians, with the exception of bureaucracy (all pair-wise Bonferroni corrected post-hoc tests *p* < 0.05).

Statistical significant differences (*p < 0.05 and **p < 0.01; respectively) between the perceived (students) and experienced (veterinarians) burden within each stressor.

**Figure 2:**
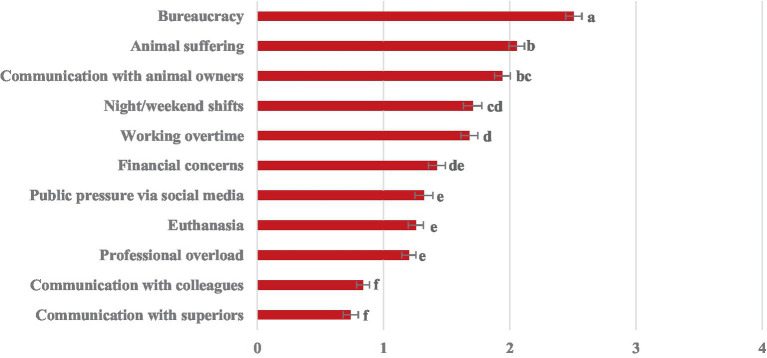
Experienced veterinary work-related stressors in veterinarians rated on a 5-point scale from 0 “not at all or not applicable” to 4 “very strongly”. Different letters (a, b, c, d, e, f) indicate statistically significant differences between stressors. Stressors with different letters are significantly different from each other (*p* < 0.05 after Bonferroni-correction).

Significant differences in stressors within the veterinarian sample are depicted in more detail in Figure 2. Bureaucracy was experienced as the most burdensome stressor, followed by animal suffering and communication with animal owners. Also, night/weekend shifts and working overtime ranked among the top 5 stressors. Financial concerns were intermediate, not differing significantly from stressors due to public pressure via social media, euthanasia, and professional overload. Communication with colleagues and superiors were rated to be the least burdensome.

Students expected animal suffering, working overtime, night/weekend shifts, communication with animal owners and financial concerns as most burdensome (Supplementary Figure S2). Bureaucratic burden was intermediate, not differing significantly from stressors due to euthanasia and public pressure via social media. The lowest burden was expected due to communication with superiors and colleagues.

**Figure 3:**
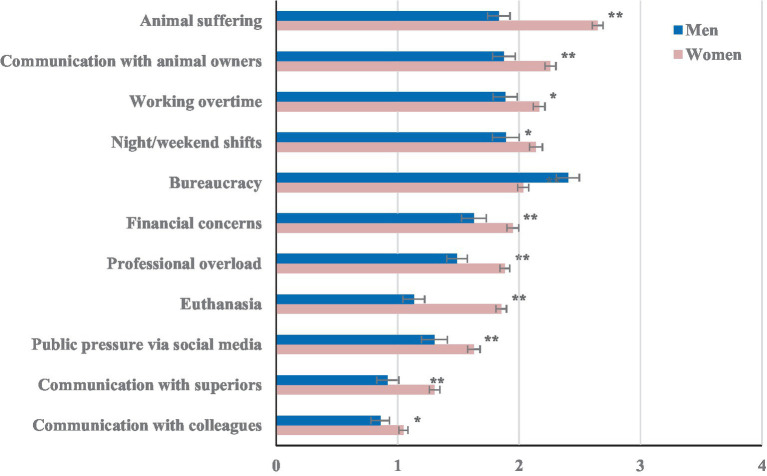
Estimated/experienced veterinary work-related stressors in female versus male veterinary students and veterinarians. The 11 pre-defined work-related stressors were rated on a 5-point scale from 0 “not at all or not applicable” to 4 “very strongly”. The stressors are listed in descending order of the females. **The difference between men and women within each stressor is significant at the 0.01 level. *The difference between men and women within each stressor is significant at the 0.05 level.

The analysis of the interaction between gender and stressor (Figure 3) shows that women indicated bureaucracy as less burdensome than men, whereas the opposite was noticed for all other work-related stressors (all pair-wise Bonferroni corrected post-hoc tests *p* < 0.05).

Bonferroni-corrected comparisons within gender are shown in more detail in Supplementary Figures S3, S4. In women, animal suffering was the top stressor, followed by communication with animal owners, working overtime and night/weekend shifts (Supplementary Figure S3). Bureaucracy, financial concerns, professional overload and euthanasia were intermediate. Communication with colleagues was rated to be the least burdensome.

Significant differences in stressors within men are depicted in more detail in Supplementary Figure S4. Bureaucracy was the main stressors, followed by night/weekend shift, working overtime, communication with animal owners and animal suffering. Euthanasia, communication with superiors and colleagues were rated as the least burdensome.

Results on the associations of occupational factors with work-related stressors are summarized in Supplementary Tables S1–S8. In brief, self-employed veterinarians experience more bureaucratic burdens but fewer issues with superiors. Veterinarians in curative practice stated to be more heavily burdened by night and weekend shifts compared to those not in curative practice. Veterinarians working with livestock stated to encounter more bureaucratic burdens (especially those working with ruminants and pigs) but to have fewer financial concerns (i.e., veterinarians working with ruminants and poultry) than those not working with these animals. Small animal practitioners seem to be particularly confronted with communication with pet owners, professional overload, financial worries, and public pressure via social media.

### 3.3 Qualitative results

#### 3.3.1 Results: practicing vets

Within the *N* = 440 veterinarians who took part in the study, 363 answered the open-ended question: “Are there other aspects that you find stressful in your work as a veterinarian? If so, please describe them.” Of these, *n* = 7 participants did not indicate any other stresses. Therefore, responses of 356 veterinarians were analyzed. Qualitative content analysis resulted in 16 main categories and 32 subcategories. All main and subcategories are shown in Table 1.

**Table 1:** Main and subcategories that emerged from the content analysis of the open-ended question: “Are there other aspects that you find stressful in your work as a veterinarian? If so, please describe them.”

Main and subcategories	*N*	%
Workplace-related stressors	93	21.1%
High workload	48	10.9%
Permanent availability	19	4.3%
High mobility	7	1.6%
Self-employment	5	1.1%
Training	4	0.9%
Heavy physical work	3	0.7%
Gender relations	2	0.5%
Career advancement	1	0.2%
Hierarchy	1	0.2%
Noise in the workplace	1	0.2%
High responsibility	1	0.2%
Technicisation of medicine	1	0.2%
Expectations of animal owners	44	10.0%
Financial aspects	40	9.1%
Settlements with animal owners	20	4.5%
Underpayment	13	3.0%
Tax/duties	4	0.9%
Cost/inflation	3	0.7%
Colleagues	28	6.4%
Expectations	12	2.7%
Uncollegiality	9	2.0%
Issues with superiors	5	1.1%
Competitive pressure	2	0.5%
Mental stressors	20	4.5%
Mental stress	12	2.7%
Self-doubt	4	0.9%
Bullying	2	0.5%
Suicide among colleagues	1	0.2%
Lack of psychological knowledge	1	0.2%
Ethical aspects	16	3.6%
Animal suffering/welfare	10	2.3%
Animal as a substitute	4	0.9%
Euthanasia	2	0.5%
Work-life-Balance	15	3.4%
Bureaucracy	13	3.0%
Professional associations	12	2.7%
Austrian Veterinary Chamber	10	2.3%
Animal health services	2	0.5%
Online platforms	10	2.3%
Negative reviews	7	1.6%
Online advisors	3	0.7%
Lack of appreciation	9	2.0%
Leading a team	5	1.1%
Shortage of skilled staff	5	1.1%
Other	4	0.9%
Politics	2	0.5%
Shortage of medication	2	0.5%

Percentages relate to the whole study sample of veterinarians who took part in the survey (*n* = 440) and not to the participants who answered the open-ended question (*n* = 356).

##### 3.3.1.1 Workplace-related stressors

The main stress factor for the veterinarians was “Workplace-related stressors,” which was mentioned by 21.1% (*n* = 93) veterinarians. This main category comprised 12 subcategories. The largest subcategory with *n* = 48 was “high workload,” as described by respondent (R) R 125: “*I worked as a vet after my studies, in 24-h clinics with night shift as well. I would not wish that time on anyone. Not knowing whether you have a holiday off or must work is stressful. Not knowing whether you can be with your family at Christmas or whether you have a 12 or even 24-h shift is very stressful. Regular working hours are rare*.” The second subcategory with *n* = 19 concerns “permanent availability” as a veterinarian. Another subcategory was “high mobility” at work mentioned by *n* = 7 vets. “Self-employment” in the veterinary profession is mentioned by *n* = 5 as a further stress factor. “Training” (0.9%, *n* = 4) as a burden was mentioned by people who either saw a discrepancy between teaching at the university and veterinary practice (R. 284) or did not have the financial means for training (R. 364). Heavy physical work mentioned by *N* = 3 was also perceived as a stress. “Gender relations” are mentioned as a pressure by *n* = 2 participants: “*But men are more likely to be protected by senior bosses than women! It’s a secondary burden that you have to be twice as good as woman, but even if you are, you are severely disadvantaged and undervalued because of your uterus and the possibility of becoming pregnant, even though you have the greatest potential and the highest education of all*!” (R. 173). “Career advancement” (*n* = 1), “Hierarchy” (*n* = 1), “Noise in the workplace” (*n* = 1), “High responsibility” (*n* = 1) and “Technicisation of medicine” (*n* = 1) were further subcategories.

##### 3.3.1.2 Expectations of animal owners

This main category subsumed the animal owners’ general expectations of veterinarians. In their responses, 10.0% (*n* = 44) described the attitude of the animal owners and the pressure or inadequacies to which they are exposed, for example R. 388: “*The implicitness with which it is expected that, as a vet, you do not refuse to help at any time, as you are unfortunately too morally obligated*”. In this context, reference is also made to the expectation of animal owners who want to relinquish responsibility but do not really co-operate with veterinarians, such as R. 244: “*That patient owners want us to relieve them of all responsibility on the one hand, but at the same time have no trust*.”

##### 3.3.1.3 Financial aspects

The third main category is “Financial aspects” (9.1%, *n* = 40), comprising of four subcategories. The majority of statements (*n* = 20) fell within the subcategory “settlements with animal owners” such as “*The conversation about costs and that patient owners keep blaming us vets*” (R. 244). The second subcategory describes “underpayment” (*n* = 13) as a veterinarian: “*Although I am always available and work as much as I can, there is never any money left over, saving is impossible*” (R. 126). Two further subcategories were “tax/duties” (*n* = 4) and “cost/inflation” (*n* = 3).

##### 3.3.1.4 Colleagues

The main category “Colleagues” with 6.4% (*n* = 28) is composed of answers dealing with the expectations of colleagues and lack of solidarity among colleagues that can be subsumed in four categories. The first subcategory with (*n* = 12) refers to the general “expectations” among colleagues at the workplace, the attitude of older colleagues towards younger colleagues or the generational conflict as described by R. 196: “*Generational conflict - older colleagues always generalize that younger colleagues do not want to work. I get this resentment too, even though I work 40 h a week and often do overtime*.” The second subcategory (*n* = 9) comprised those responses that indicated “uncollegiality” in the veterinarians’ working environment. The subcategory “Issues with superiors” with 1.1% (*n* = 5) consisted of responses that referred to conflicts with managers. The last subcategory is “competitive pressure” among colleagues with *n* = 2.

##### 3.3.1.5 Mental stressors

The main category “Mental stressors,” with *n* = 20 (4.5%) responses, refers to psychological and emotional pressure from working as a veterinarian and was summarized into five subcategories. The first subcategory, with *n* = 12, is “mental stress” outside of working hours, such as R. 169 emphasized: “*not being able to switch off in your free time* … *the feeling of being “alone”* …” In the “self-doubt” subcategory (*n* = 4), participants stated that they felt they were not good enough for the job and had doubts about their own professional performance: “*Self-doubt that the animal would have been treated better elsewhere. Feelings of guilt that the animal has now ended up with me. Questioning after the working day whether I have really done everything possible. Fear of having forgotten something*” (R. 184). The three other subcategories were “Bullying” (*n* = 2), “Suicide among colleagues” (*n* = 1) and “Lack of psychological knowledge” (*n* = 1).

##### 3.3.1.6 Ethical aspects

A total of *n* = 16 (6.3%) respondents reported further stressors related to ethical aspects. This main category comprised three subcategories. The subcategory of “Animal suffering/welfare” (2.3%, *n* = 10) as a stress factor is made up of responses that are confronted with animal distress in the course of their veterinary work, which could be caused by neglect on the part of animal owners. The subcategory “animal as a substitute” (0.9%, *n* = 4) was an indication of the role of the animal as a substitute for a child or partner that was not fulfilled. The subcategory of “euthanasia” was emphasized by 0.5% (*n* = 2) of participants as a stress factor.

##### 3.3.1.7 Work-life-balance

The next main category was the “Work-life-balance” (3.4%, *n* = 15). For example, R. 282 wrote: “*With family routines, possibly planning holidays, or the feeling of not having enough time for partner and children*.”

##### 3.3.1.8 Bureaucracy

The main category of “Bureaucracy” was mentioned by 3.0% (*n* = 13) respondents. For example, respondent 124 wrote that there was little time left for work because of bureaucratic regulations: “*Time for actual veterinary work is becoming increasingly scarce due to unnecessary and unpaid bureaucratic tasks that have nothing to do with veterinary medicine*.”

##### 3.3.1.9 Professional associations

Another main category concerns the “Professional associations” with 2.7% (*n* = 12) responses. The first subcategory is the “Austrian Veterinary Chamber” with *n* = 10. Respondent 34 expressed his dissatisfaction as follows: “*Lack of support from the Austrian Veterinary Chamber with regard to legal certainty in the practice of the profession*.” The next subcategory was “animal health services” (*n* = 2), referring to governmental institutions responsible for monitoring and promoting animal health and implementing disease control measures.

##### 3.3.1.10 Online platforms

The main category “Online platforms” (2.3%, *n* = 10) refers to anonymous ratings and reviews online such as Google and also to guides on the internet. This main category comprised of two subcategories: “Negative reviews” with *n* = 7 and “online advisors” with *n* = 3.

##### 3.3.1.11 Further main categories

The main category “Lack of appreciation” with 2.0% (*n* = 9) referred to a disregard and lack of acceptance of the veterinarians’ contributions to society and to animal owners. The main category of “Leading a team” (1.1%, *n* = 5) refers to aspects of stress associated with leadership tasks. Another main category was the “Shortage of skilled staff” with 1.1% (*n* = 5). All responses that could not be assigned to the main or subcategories were subsumed in the main category “Other” (0.9%, *n* = 4). The main category of “Politics” (0.5%, *n* = 2) is made up of very short answers on general politics as a stress factor. The main category “Shortage of medication” is also made up of 0.5% (*n* = 2) responses.

#### 3.3.2 Results: veterinary students

A total of *N* = 430 veterinary students participated in the study. *N* = 122 of the participants answered the open-ended question: “Are there other aspects of your work as a veterinarian that you consider stressful? If yes, please describe which ones.” Of these, *n* = 3 participants did not report any other stressors and 3 participants will not be practicing veterinary medicine after graduation. The responses of 116 participants were analyzed and resulted in 10 main categories and 30 subcategories (Table 2). The results of the main and subcategories of content analysis are described below.

**Table 2:** Main and subcategories that emerged from the content analysis of the students’ open-ended question: “Are there other aspects of working as a veterinarian that you consider stressful? If yes, please describe which ones?”

Main and subcategories	*n*	%
Workplace-related stressors	51	11.9%
Working conditions	19	4.4%
Stress and pressure to perform	11	2.6%
Responsibility	9	2.1%
Gender relations	7	1.6%
Competitive pressure	4	0.9%
Continuing education	3	0.7%
Education-career transition	2	0.5%
Career choice	1	0.2%
Work-life-balance	50	11.6%
Compatibility of family and career	27	6.3%
Work-life preference	23	5.3%
Mental stressors	32	7.4%
Self-doubt	10	2.3%
Anxiety	10	2.3%
Mental stress	5	1.2%
Uncertainty	4	0.9%
Burnout	1	0.2%
Well-being	1	0.2%
Boringness	1	0.2%
Financial aspects	29	6.7%
Underpayment	15	3.5%
Financial issues with animal owner	9	2.1%
Veterinary association fees	2	0.5%
Lack of compensation	1	0.2%
Inflation	1	0.2%
Insurance costs	1	0.2%
Communication with animal owners	10	2.3%
Ethical aspects	6	1.4%
Animal suffering	2	0.5%
Ethical considerations	2	0.5%
Animal as a substitute	1	0.2%
Compassion for animals	1	0.2%
Lack of appreciation	5	1.2%
Public pressure	4	0.9%
Bureaucracy	4	0.9%
Official veterinarians	2	0.5%
Legislative problems	1	0.2%
Bureaucratic hurdles	1	0.2%
Other	3	0.7%

Percentages relate to the whole study sample of veterinary students who took part in the survey (*n* = 430) and not to the participants who answered the open-ended question (*n* = 116).

##### 3.3.2.1 Workplace-related stressors

The most frequently mentioned stress factor for veterinary students was “workplace-related stressors,” described by *n* = 51 (11.9%) of respondents. The largest subcategory with *n* = 19 was “working conditions” and included responses such as general workload, irregular shifts or the general “*amount of work due to a shortage of veterinarians*” (R. 184). The second subcategory, with *n* = 11, concerns “stress and pressure to perform” directly related to working conditions. The third subcategory is made up of responses (with *n* = 9) that describe “responsibility” and was specifically taking care of the life and welfare of animals. “Gender relations” at work was mentioned as a pressure by *n* = 7 participants: “*Gaining the respect of customers as a woman*” (R. 65). “Competitive pressure” with *n* = 4 answers showed a high level of agreement both among students and in order to get a suitable training place. R. 219 addressed it as follows: “*Increasing competitive pressure among students during their studies and the associated decrease in willingness to help each other*.” The subcategory “continuing education” (*n* = 3) refers to those responses that considered compulsory continuing education during their employment to be a burden. The category “education-career transition” (*n* = 2) indicated a stress factor related to starting work after leaving university. Other subcategories were “education-career transition” (*n* = 2), and “career choice” with *n* = 1.

##### 3.3.2.2 Work-life-balance

The main category of “work-life-balance” (*n* = 50, 11.6%) included stressors that result from an interaction between the person and the environment and influence the experience of balance. The first subcategory was “compatibility of family and career” (*n* = 27) and signaled the difficulty of starting a family as a veterinarian. For example, R. 135 described “*in livestock farming, the vet has to prioritize his profession over his private life*.” The second subcategory, “work-life preference” mentioned by *n* = 23 participants, indicated either a lack of balance between work and private life or concern about the possibility of achieving a balance at all. Another aspect related to the shortage of leisure time for social activities.

##### 3.3.2.3 Mental stressors

The main category “Mental stressors,” with *n* = 32 (7.4%) responses, related to the psychological and emotional aspects of working as a veterinarian. In the first subcategory “self-doubt” (*n* = 10), students stated that they feared that they would not be well qualified for the practical side of the profession after graduation: “*When I graduate as a vet, I’ve already heard that I’ll get my own patients from day one, even though I have no idea. This really gets me down and I’m thinking of changing to a profession where you have less responsibility, I’m afraid of failing*” (R. 239). The second subcategory labeled “anxiety” (*n* = 10) refers to worries about making professional mistakes due to time pressure and stress. The third subcategory, “mental stress” (*n* = 5), referred to psychological stresses that arose during the study and caused general concern, such as “*depression and suicide rates among veterinarians*” (R. 218). The subcategory “uncertainty” (*n* = 4) referred to responses that perceived lack of knowledge and helplessness in practice as a stressor. The other three subcategories were “burnout” (*n* = 1), “well-being” (*n* = 1) and “boringness” (*n* = 1).

##### 3.3.2.4 Financial aspects

The main category “financial aspects,” mentioned by *n* = 29 (6.7%) participants, consisted of different aspects directly related to financial concerns. The first subcategory “underpayment,” with *n* = 15 responses, indicated the link between high workload and low pay in the profession. The second subcategory, “Financial issues with animal owners” (*n* = 9), highlights concerns about the financial aspects of animal treatment that owners do not provide: “… *having to talk to pet owners about money and sometimes not being able to do proper treatments because they are too expensive* …” (R. 105). Other subcategories are “veterinary association fees” (*n* = 2), “lack of compensation” (*n* = 1), “inflation” (*n* = 1) and “insurance costs” (*n* = 1).

##### 3.3.2.5 Communication with animal owners

The main category “communication with animal owners,” mentioned by *n* = 10 (2.3%) respondents, indicates stress factors in the interaction between animal owners and veterinarians, as there are different expectations. R. 155 expressed it as follows: “*Reconciling the welfare of the animal with the wishes of the owner*.”

##### 3.3.2.6 Ethical aspects

The main category “Ethical aspects” with *n* = 6 (1.4%) was based on the following subcategories: “animal suffering” (*n* = 2), “ethical considerations” (*n* = 2), “compassion for animals” (*n* = 1) and “animal as a substitute” (*n* = 1) to replace a missing partner or child.

##### 3.3.2.7 Lack of appreciation

The main category “lack of appreciation” (*n* = 5) referred to stress factors related to the general acceptance of veterinary services in society, i.e., a veterinarian is not recognized as much as a human doctor (R. 173 and 203).

##### 3.3.2.8 Further main categories

The main category “public pressure” (*n* = 4, 0.9%) consisted of responses that identified the public’s expectations of veterinarians as a stress factor. The main category “bureaucracy” (*n* = 4, 0.9%) was composed of three subcategories, which were either administrative worries with “official veterinarians” (*n* = 2) or “legislative problems” (*n* = 1) or “bureaucratic hurdles” (*n* = 1) as a future burden in the profession. All responses from students that could not be assigned to the main or subcategories were included in the category “Other” (*n* = 3, 0.7%).

### 3.4 Associations between perceived occupational stress factors in veterinarians and indicators of mental health

Table 3 illustrates correlations observed between mental health indicators in veterinarians and the extent of experienced stressors among the 11 pre-defined occupational stressors.

**Table 3:** Pearson correlation analyses investigating associations of the experienced burden of work-related stressors and indicators of mental health in Austrian veterinarians (*N* = 440).

Stressor	Depression (PHQ-9)	Anxiety (GAD-7)	Insomnia (ISI-2)	Well-being (WHO-5)	Stress (PSS-4)
Communication with animal owners	0.389*	0.391*	0.301*	−0.319*	0.351*
Communication with colleagues	0.335*	0.347*	0.206*	−0.242*	0.316*
Communication with superiors	0.320*	0.297*	0.213*	−0.242*	0.283*
Night/weekend shifts	0.369*	0.310*	0.243*	−0.339*	0.314*
Working overtime	0.373*	0.370*	0.277*	−0.374*	0.346*
Euthanasia	0.335*	0.326*	0.277*	−0.200*	0.261*
Animal suffering	0.316*	0.276*	0.257*	−0.217*	0.267*
Bureaucracy	0.164*	0.141	0.206*	−0.176*	0.212*
Professional overload	0.385*	0.394*	0.258*	−0.291*	0.322*
Financial concerns	0.461*	0.473*	0.361*	−0.375*	0.426*
Public pressure via social media	0.314*	0.327*	0.258*	−0.214*	0.325*

*The correlation is significant after correcting for multiple testing (*p* < 0.05/55 correlation analyses). Work-related stressors were rated on a 5-point Likert scale ranging from 0 “not at all/not applicable” to 4 “very strongly”.

Strongest associations of perceived stress and mental health indicators were observed for financial concerns, with Pearson correlation coefficients ranging from *r* = 0.36 to *r* = 0.47. Also, the perceived stress through communication with animal owners showed a moderate correlation with all investigated mental health parameters (*r* between 0.30 and 0.39). The extent to which night/weekend shifts and working overtime were perceived as stressful were also moderately associated with symptoms of depression, anxiety, well-being and stress (*r* between 0.31 and 0.37). The stressor with the weakest association with mental health indicator was bureaucracy (*r* between 0.14 and 0.21).

Associations of expected stress and actual mental health indicators in students are summarized in Supplementary Table S9, revealing only weak associations (*r* between 0.03 and 0.29). Interpreting these results must be approached with caution, as it’s intricate to establish a direct relationship between current emotional states and future stressors.

## 4 Discussion

One major finding of the present study elucidates that aspiring veterinarians exhibit a notable awareness of the substantial stress associated with their desired profession. Remarkably, their perceptions consistently exceed the experienced stress levels in all domains, except for administrative stressors. This empirical evidence substantiates that there is no need to further highlight the already acknowledged occupational stress. Instead, it underscores the paramount need for targeted interventions aimed at imparting requisite coping skills to students. Such interventions are indispensable for equipping these future professionals with the proficiency to navigate their forthcoming careers adeptly, consequently mitigating the risk of psychological overload and premature career attrition. These results not only contribute to the scholarly understanding of veterinary education but also hold practical implications for the development of tailored interventions and support mechanisms in this professional domain.

A further main finding is that Austrian veterinarians identified bureaucracy as the most onerous factor from a predefined list of 11 potential stressors. Additionally, studies conducted in Belgium and the UK (12, 15) revealed that administrative formalities were consistently rated as highly stressful within the realm of veterinary practice. However, these prior investigations did not establish a direct link between this administrative burden and its impact on mental health indicators. In our own study, while bureaucracy similarly emerged as the predominant stressor within the veterinary profession, we found that its correlations with perceived stress levels and insomnia were small (*r* = 0.21), with negligible associations observed in relation to other health indicators (*r* < 0.20). This suggests that other factors, not directly related to administrative burdens, may play a more significant role in contributing to the overall stress and well-being of veterinary professionals. Moreover, it’s worth noting that dealing with bureaucracy is not a skill veterinarians are explicitly trained for or aim to do in their daily practice. It is not the primary focus of their veterinary training. The bureaucratic tasks keep veterinarians from focusing on what they were trained for, adding to their stress levels. This may explain why bureaucracy is not highlighted as a significant stressor by students, as they enter the profession unprepared for the administrative burden of legal formalities, managing orders, taxes, and other bureaucratic tasks.

Contrary to administrative duties, financial worries did not rank among the top job-related concerns of Austrian veterinarians; nevertheless, they exhibited the strongest negative association with mental health. These findings align with research conducted in the Austrian general population, which indicated the highest prevalence of mental illness symptoms within the low-income group (34). Notably, previous studies in the United States have highlighted financial concerns as significant occupational stressors linked to psychological distress in veterinarians (19, 35). However, direct comparisons with these studies are complex due to variations in the educational systems and financial burdens encountered. It is pertinent to recognize that in Austria, most degrees, including veterinary medicine, are publicly funded, with students generally not incurring tuition fees for their education. The results of the current study underscore the significance of low compensation within the veterinary profession as the most substantial factor associated with poor mental health among veterinarians. Given the substantial stress levels and the tangible influence of financial pressures, seeking guidance from certified financial planners to establish sustainable strategies for managing living expenses within income constraints could prove to be a beneficial avenue for addressing this critical issue already during education. In addition to addressing individual financial concerns, it’s crucial to acknowledge the systemic issues within the veterinary profession that contribute to financial stress. For instance, the fact that veterinarians are often paid by other veterinarians may lead to internal conflicts over fair compensation and recognition of financial burdens. Moreover, the implementation of fee regulations, as seen in Germany, could prevent price undercutting and provide veterinarians with more stability in their earnings. Furthermore, systemic factors such as economic conditions, political decisions, and market forces, particularly in agricultural settings, significantly impact how much clients are willing to pay for veterinary services. By considering these systemic challenges and advocating for structural changes, we can address the root causes of financial stress among veterinarians, going beyond individual financial management strategies.

Results on high perceived burden through conversation with animal owners as well as the association of this perceived burden with mental health indicators is in line with previous studies conducted in the US, UK, Belgium, and Germany ranking client relations among the most stressful factors identified by veterinarians (5, 12, 15, 19, 35). More specifically, dealing with clients was stated to be burdensome due to client complaints, dealing with client grief, client expectations, lack of respect, ungratefulness, later/unpaid invoices, and phone harassment during practice. Indeed, also answers to the open-ended question on further perceived job-related worries in the study at hand revealed several stressors related to client expectations, such as being accessible around the clock or worries related to treatment costs. The perception of clients who view animals as “objects” or as “substitutes for human relationships (partners/children)” is also regarded as burdensome. In Austria, veterinarians have historically not received specialized training for interacting with people. It’s only in recent years that veterinary students have been introduced to a course on “client conversation” as part of their curriculum. It is important to acknowledge that individuals who choose to study veterinary medicine possess specific cognitive and personality traits (7). Subconsciously, the decision to pursue a veterinary career may be influenced by a preference for working with animals rather than people, which could consequently impact their perception of client interactions as burdensome. Moreover, veterinary students are often under high academic pressure, facing a demanding curriculum and intense competition, which may hinder the development of their social and communication skills (7).

Animal suffering ranked among the most stressful factors reported in the current study. A unique challenge in veterinary practice is the dependency of the animals’ well-being on the owners’ decisions and financial situation. The animal owner might for instance decide against veterinary advice in terms of treatment (e.g., if they cannot afford the treatment costs, or refuse euthanasia for a pet “acting as a surrogate child” due to emotional attachment despite severe health issues) but also in terms of other aspects such as housing or breeding. Although in Europe there are official regulations regarding how the different animal species should be housed, there are many animal owners who do not stick to the rules, are not aware, or do not care how and if their animal is kept up to its natural needs. Another veterinary-specific issue pertains to breeding practices aimed at achieving certain aesthetic standards, often at the expense of animal welfare and health. The human ideas of an “ideal” individual within a breed has led to the breeding of various races within dogs, cats, and cattle, with a predisposition to numerous health issues and syndromes. Unfortunately, many animal owners fail to grasp the physical discomfort experienced by their animals due to genetic factors. This lack of awareness often results in the neglected veterinary advice and the perpetuation of breeding practices that prioritize aesthetics over the well-being of the animals involved (21). All these circumstances put ethical pressure on the vet, often recommendations of veterinarians to client owners are not followed, making the situation for the animal more miserable.

A further stressor, mostly unique for veterinarians, is performing euthanasia. However, neither in our study, nor in previous studies did euthanasia rank among the top 5 stressors reported by veterinarians (15, 18). As animal suffering, or the grief of clients or the staff due to animal illness or euthanasia are ranked more frequently as stressors, findings could suggest that not the death of the animals itself is putting most stress on veterinarians, but rather the intense emotional distress experienced by the humans involved. Further open questions revealed that ethical pressure, when the owner has no money or bad compliance to treat the animal, is rather a stressor than euthanasia *per se*.

Furthermore, trait perfectionism, i.e., the tendency to have very high and rigid standards for the self and/or others, as an individual difference, increases vulnerability to experiencing heightened distress when confronted with morally challenging situations in veterinary practice (20). As morally significant stressors on their own tend to elicit only mild distress, the individual’s personality traits might have a more significant predictive role in job-related stress than the work environment itself (36).

Overall, our findings point out that the aspects of social interaction and ethical concerns are detrimental risk factors. The situation is further compounded by the observation that as working hours increase, the capacity for empathic and compassionate interactions, especially with grieving client owners, diminishes (37).

In line with previous studies, we observed a high perceived burden through night/weekend shifts and working overtime. Studies conducted in Australia, Belgium, Finland, the UK, Germany and New Zealand observed that the frequent overtime, on-call duties and weekend service represented one of the main stressors in the veterinary profession (5, 12–16). The top number one source of stress given by practice owners, practice associates, and relief vets was the demands of practice, e.g., long working hours, work overload (35) and poor work-life balance were reported to be the top reason to leave the veterinary profession (5). High working hours are at the expense of leisure time activities, such as engaging in physical activity and social relationships. Therefore, results of the present study are in line with our previous results, showing increased mental health burden in veterinarians who are physically inactive outside their professional activities (10). As supported by the correlation analyses, a poor work-life balance can lead to diminished mental health. Free text answers to the open-ended question on other work-related perceived stress factors revealed that a poor work-life balance was a significant stressor for veterinarians. They reported a lack of compatibility between their professional and personal life, particularly when it comes to fulfilling family responsibilities alongside work commitments. Overall, the analysis of free-text responses of students regarding aspects expected to be stressful in their future careers as veterinarians indicates that students primarily fear poor work-life balance and difficulties in reconciling their profession with family life. These results, coupled with the generally higher perceived work-related stress levels by students compared to the veterinarians, may suggest that the demanding workload experienced by Austrian veterinarians could be a major contributing factor to premature attrition from the profession. It’s worth noting that the sample of veterinarians primarily includes those who have chosen to remain in the profession, rather than those who have already pursued different career paths due to the substantial stress they experienced. Therefore, it can be concluded that lower job-related demands enabling veterinarians to allocate time for family-related activities and obligations, time for real social relationships, support from friends, partners, and families would help to improve resilience of veterinarians against mental disruptions (5, 22). Therefore, the work-life balance, focusing on nurturing social ties and recreational physical activity should be addressed in preventive programs for both, students, and practising veterinarians.

In our survey, some participants highlighted challenges related to gender issues as a notable concern. For instance, several veterinary students expressed concerns about gaining respect from customers as women, suggesting a perceived gender bias in client interactions. Additionally, some comments from students and veterinarians underscored broader systemic issues, with some noting disparities in how men and women are treated within the profession. Some veterinarians reported additional pressure felt by women, highlighting the need to surpass higher standards and the persistent undervaluation they face due to gender stereotypes, including concerns related to pregnancy. Notably, our survey sample reflected a higher proportion of women, both among students (85.8%) and veterinarians (72%), which underscores a gender imbalance within the field. These findings suggest a need for further exploration of gender dynamics and support mechanisms within the veterinary profession to address underlying inequalities and promote inclusivity.

The overall higher burden anticipated/experienced by female students/veterinarians is in line with a recent scoping review, highlighting that female veterinarians perceive a higher psychological workload compared to their male counterparts (5). In line with these findings, female students and veterinarians participating in the current study experienced a higher mental health burdened compared to their male colleagues (9, 10).

This study has some limitations. First, there’s a significant potential for nonresponse bias, which may lead to either an overestimation or underestimation of the estimated/experienced stressors related to veterinary practice. It remains unclear whether students and veterinarians experiencing profound psychological distress were more inclined to participate in the questionnaire due to their vested interest in the topic or, conversely, less inclined due to factors such as reduced interest or energy, or social withdrawal. Second, all mental health indicators were based on self-reports and not confirmed by structured clinical or standardized interviews due to the online nature of the study. Third, the cross-sectional design of the study does not allow for causal conclusions. Lastly, it is worth noting that the questionnaire lacked inquiries designed to evaluate personality traits, family histories of mental illness, or other factors that could conceivably impact the perception of job-related stressors as well as mental health.

## 5 Conclusion

Results suggest that poor mental health in Austrian veterinarians is mainly associated with perceived financial worries, communication with clients and high workload. Comparisons between data from veterinary students and practicing veterinarians suggest that professional bodies and veterinary universities can play a vital role in raising awareness among students and veterinarians about the significance of mental health and overall well-being, while also encouraging them to allocate time for self-care activities. Implementing measures to limit excessive work hours and evening shifts could also prove beneficial. Considering the significant stress levels inherent in the veterinary profession and the evident impact of financial pressures, we recommend that veterinarians consider seeking guidance to develop a stress management strategy aimed at enhancing their stress-coping abilities and consider consultations with mental health and financial planning experts.

## Data availability statement

The raw data supporting the conclusions of this article will be made available by the authors, without undue reservation.

## Ethics statement

The studies involving humans were approved by Ethics committee of the University for Continuing Education Krems. The studies were conducted in accordance with the local legislation and institutional requirements. The participants provided their electronic informed consent to participate in this study.

## Author contributions

VN: Conceptualization, Data curation, Investigation, Methodology, Supervision, Writing – original draft. AG: Formal Analysis, Investigation, Methodology, Software, Writing – original draft. TP: Writing – review & editing. DB: Conceptualization, Data curation, Methodology, Writing – review & editing. RD: Conceptualization, Methodology, Writing – review & editing. CP: Writing – review & editing. EH: Conceptualization, Data curation, Formal Analysis, Investigation, Methodology, Project administration, Resources, Software, Supervision, Validation, Visualization, Writing – original draft.

## References

[1] Cox R. Some problems and possibilities of caring. Ethics Place Environ. (2010) 13:113–30. doi: 10.1080/13668791003778800

[2] Donald MM. When care is defined by science: exploring veterinary medicine through a more-than-human geography of empathy. Area. (2019) 51:470–8. doi: 10.1111/area.12485

[3] Magalhães-Sant‘Ana M Lassen J Millar KM Sandøe P Olsson IAS. Examining why ethics is taught to veterinary students: a qualitative study of veterinary educators’ perspectives. J Vet Med Educ. (2014) 41:350–7. doi: 10.3138/jvme.1113-149R 24816827

[4] Österreich Bundeskanzleramt. RIS—Tierärztegesetz - Bundesrecht konsolidiert, Fassung vom 07.08.2023. (2018) https://www.ris.bka.gv.at/GeltendeFassung.wxe?Abfrage=Bundesnormen&Gesetzesnummer=20011642 (Accessed August 7, 2023)

[5] Pohl R Botscharow J Böckelmann I Thielmann B. Stress and strain among veterinarians: a scoping review. Ir Vet J. (2022) 75:15. doi: 10.1186/s13620-022-00220-x, PMID: 35729648 PMC9209636

[6] Brscic M Contiero B Schianchi A Marogna C. Challenging suicide, burnout, and depression among veterinary practitioners and students: text mining and topics modelling analysis of the scientific literature. BMC Vet Res. (2021) 17:294. doi: 10.1186/s12917-021-03000-x, PMID: 34488757 PMC8419380

[7] Bartram DJ Baldwin DS. Veterinary surgeons and suicide: a structured review of possible influences on increased risk. Vet Rec. (2010) 166:388–97. doi: 10.1136/vr.b4794 20348468

[8] Zimmermann C Strohmaier S Niederkrotenthaler T Thau K Schernhammer E. Suicide mortality among physicians, dentists, veterinarians, and pharmacists as well as other high-skilled occupations in Austria from 1986 through 2020. Psychiatry Res. (2023) 323:115170. doi: 10.1016/j.psychres.2023.115170, PMID: 37001488

[9] Humer E Neubauer V Brühl D Dale R Pieh C Probst T. Prevalence of mental health symptoms and potential risk factors among Austrian veterinary medicine students. Sci Rep. (2023) 13:13764. doi: 10.1038/s41598-023-40885-0, PMID: 37612368 PMC10447431

[10] Neubauer V Dale R Probst T Pieh C Janowitz K Brühl D. Prevalence of mental health symptoms in Austrian veterinarians and examination of influencing factors. Sci Rep. (2024) 10.1038/s41598-024-64359-z PMC11164977 38858563

[11] Dale R Humer E Probst T Brühl D Pieh C Wenninger O-D. Risk factors for suicidality in veterinarians in Austria. Response to the article entitled: suicide mortality among physicians, dentists, veterinarians, and pharmacists as well as other high-skilled occupations in Austria from 1986 through 2020. Psychiatry Res. (2023) 327:115410. doi: 10.1016/j.psychres.2023.115410 37611326

[12] Bartram DJ Yadegarfar G Baldwin DS. A cross-sectional study of mental health and well-being and their associations in the UK veterinary profession. Soc Psychiatry Psychiatr Epidemiol. (2009) 44:1075–85. doi: 10.1007/s00127-009-0030-8, PMID: 19294320

[13] Fritschi L Morrison D Shirangi A Day L. Psychological well-being of Australian veterinarians. Aust Vet J. (2009) 87:76–81. doi: 10.1111/j.1751-0813.2009.00391.x, PMID: 19245615

[14] Gardner D Hini D. Work-related stress in the veterinary profession in New Zealand. N Z Vet J. (2006) 54:119–24. doi: 10.1080/00480169.2006.36623, PMID: 16751842

[15] Hansez I Schins F Rollin F. Occupational stress, work-home interference and burnout among Belgian veterinary practitioners. Ir Vet J. (2008) 61:233–41. doi: 10.1186/2046-0481-61-4-233, PMID: 21851711 PMC3113869

[16] Reijula K Räsänen K Hämäläinen M Juntunen K Lindbohm M-L Taskinen H. Work environment and occupational health of Finnish veterinarians: veterinarians’ work environment and health. Am J Ind Med. (2003) 44:46–57. doi: 10.1002/ajim.10228, PMID: 12822135

[17] Hagen JR Weller R Mair TS Kinnison T. Investigation of factors affecting recruitment and retention in the UK veterinary profession. Vet Rec. (2020) 187:354–4. doi: 10.1136/vr.106044, PMID: 32817568

[18] Nett RJ Witte TK Holzbauer SM Elchos BL Campagnolo ER Musgrave KJ. Prevalence of risk factors for suicide among veterinarians—United States, 2014. Morb Mortal Wkly Rep. (2015) 64:131–2. PMID: 25674997 PMC4584691

[19] Volk JO Schimmack U Strand EB Lord LK Siren CW. Executive summary of the Merck animal health veterinary wellbeing study. J Am Vet Med Assoc. (2018) 252:1231–8. doi: 10.2460/javma.252.10.1231, PMID: 29701527

[20] Crane M Phillips J Karin E. Trait perfectionism strengthens the negative effects of moral stressors occurring in veterinary practice. Aust Vet J. (2015) 93:354–60. doi: 10.1111/avj.12366, PMID: 26412116

[21] Sonntag Q Overall KL. Key determinants of dog and cat welfare: behaviour, breeding and household lifestyle. Rev Sci Tech OIE. (2014) 33:213–20. doi: 10.20506/rst.33.1.2270, PMID: 25000794

[22] Shirangi A Fritschi L Holman C Morrison D. Mental health in female veterinarians: effects of working hours and having children. Aust Vet J. (2013) 91:123–30. doi: 10.1111/avj.12037 23521096

[23] Tomlin JL Brodbelt DC May SA. Veterinary students’ understanding of a career in practice. Vet Rec. (2010) 166:781–6. doi: 10.1136/vr.b4842, PMID: 20562377

[24] Brähler E Mühlan H Albani C Schmidt S. Teststatistische Prüfung und Normierung der deutschen Versionen des EUROHIS-QOL Lebensqualität-Index und des WHO-5 Wohlbefindens-Index. Diagnostica. (2007) 53:83–96. doi: 10.1026/0012-1924.53.2.83

[25] Cohen S., Perceived stress in a probability sample of the United States, The social psychology of health. The Claremont symposium on applied social psychology. Thousand Oaks, CA, US: Sage Publications, Inc. (1988). 31–67

[26] Spitzer RL Kroenke K Williams JB. Validation and utility of a self-report version of PRIME-MDThe PHQ primary care study. JAMA. (1999) 282:1737–44. doi: 10.1001/jama.282.18.1737, PMID: 10568646

[27] Kroenke K Spitzer RL Williams JBW. The PHQ-9: validity of a brief depression severity measure. J Gen Intern Med. (2001) 16:606–13. doi: 10.1046/j.1525-1497.2001.016009606.x, PMID: 11556941 PMC1495268

[28] Spitzer RL Kroenke K Williams JBW Löwe B. A brief measure for assessing generalized anxiety disorder: the GAD-7. Arch Intern Med. (2006) 166:1092. doi: 10.1001/archinte.166.10.1092 16717171

[29] Löwe B Decker O Müller S Brähler E Schellberg D Herzog W. Validation and standardization of the generalized anxiety disorder screener (GAD-7) in the general population. Med Care. (2008) 46:266–74. doi: 10.1097/MLR.0b013e318160d093, PMID: 18388841

[30] Morin CM Belleville G Bélanger L Ivers H. The insomnia severity index: psychometric indicators to detect insomnia cases and evaluate treatment response. Sleep. (2011) 34:601–8. doi: 10.1093/sleep/34.5.601, PMID: 21532953 PMC3079939

[31] Kraepelien M Blom K Forsell E Hentati Isacsson N Bjurner P Morin CM. A very brief self-report scale for measuring insomnia severity using two items from the insomnia severity index - development and validation in a clinical population. Sleep Med. (2021) 81:365–74. doi: 10.1016/j.sleep.2021.03.003, PMID: 33813233

[32] Hsieh H-F Shannon SE. Three approaches to qualitative content analysis. Qual Health Res. (2005) 15:1277–88. doi: 10.1177/1049732305276687 16204405

[33] Atlas.ti, ATLAS.ti: The Qualitative Data Analysis & Research Software (version 8) (Computer Software). (2018), Available at: https://atlasti.com/de

[34] Humer E Schaffler Y Jesser A Probst T Pieh C. Mental health in the Austrian general population during COVID-19: cross-sectional study on the association with sociodemographic factors. Front Psych. (2022) 13:943303. doi: 10.3389/fpsyt.2022.943303, PMID: 36506423 PMC9729349

[35] Nett RJ Witte TK Holzbauer SM Elchos BL Campagnolo ER Musgrave KJ. Risk factors for suicide, attitudes toward mental illness, and practice-related stressors among US veterinarians. J Am Vet Med Assoc. (2015) 247:945–55. doi: 10.2460/javma.247.8.945, PMID: 26421408

[36] Dawson BFY Thompson NJ. The effect of personality on occupational stress in veterinary surgeons. J Vet Med Educ. (2017) 44:72–83. doi: 10.3138/jvme.0116-020R, PMID: 28206844

[37] Dow M Chur-Hansen A Hamood W Edwards S. Impact of dealing with bereaved clients on the psychological wellbeing of veterinarians. Aust Vet J. (2019) 97:382–9. doi: 10.1111/avj.12842, PMID: 31364771

