# Supplementary material for: Stress factors in veterinary medicine—a cross-sectional study among veterinary students and practicing vets in Austria
Source: Front Vet Sci. 2024 May 30;11:1389042. doi: 10.3389/fvets.2024.1389042 (PMC11169866; doi:10.3389/fvets.2024.1389042)
Supplement: Supplementary file 1 [file Data_Sheet_1.pdf]

Supplementary Material

1.1 Supplementary Figures

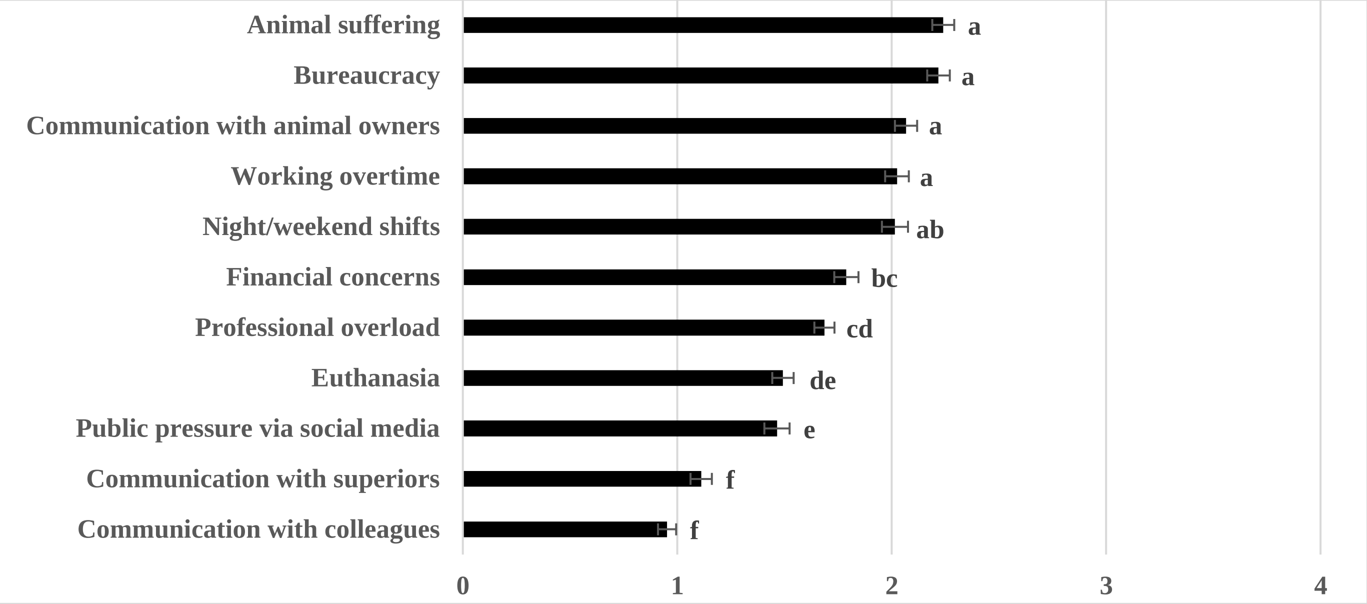

Suppl. Figure 1 Estimated/experienced veterinary work-related stressors in veterinary students and veterinarians. The 11 pre-defined work-related stressors were rated on a 5-point scale from 0 ‘not at all or not applicable’ to 4 ‘very strongly’. Different letters (a,b,c,d,e,f) indicate statistically significant differences between stressors. Stressors with different letters are significantly different from each other ($P < 0.05$ after Bonferroni-correction).

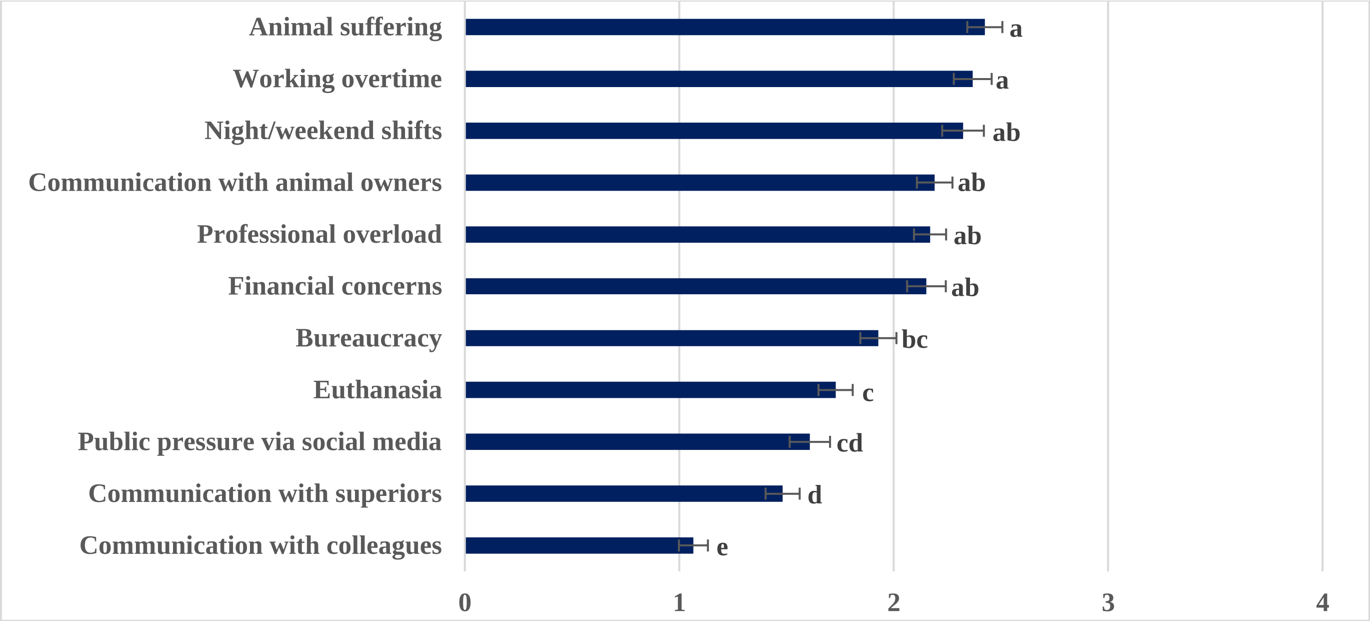

Supplementary Figure 2 Estimated veterinary work-related stressors of veterinary students rated on a 5-point scale from 0 ‘not at all’ to 4 ‘very strongly’. Different letters (a,b,c,d,e) indicate statistically significant differences between stressors. Stressors with different letters are significantly different from each other ($P < 0.05$ after Bonferroni-correction).

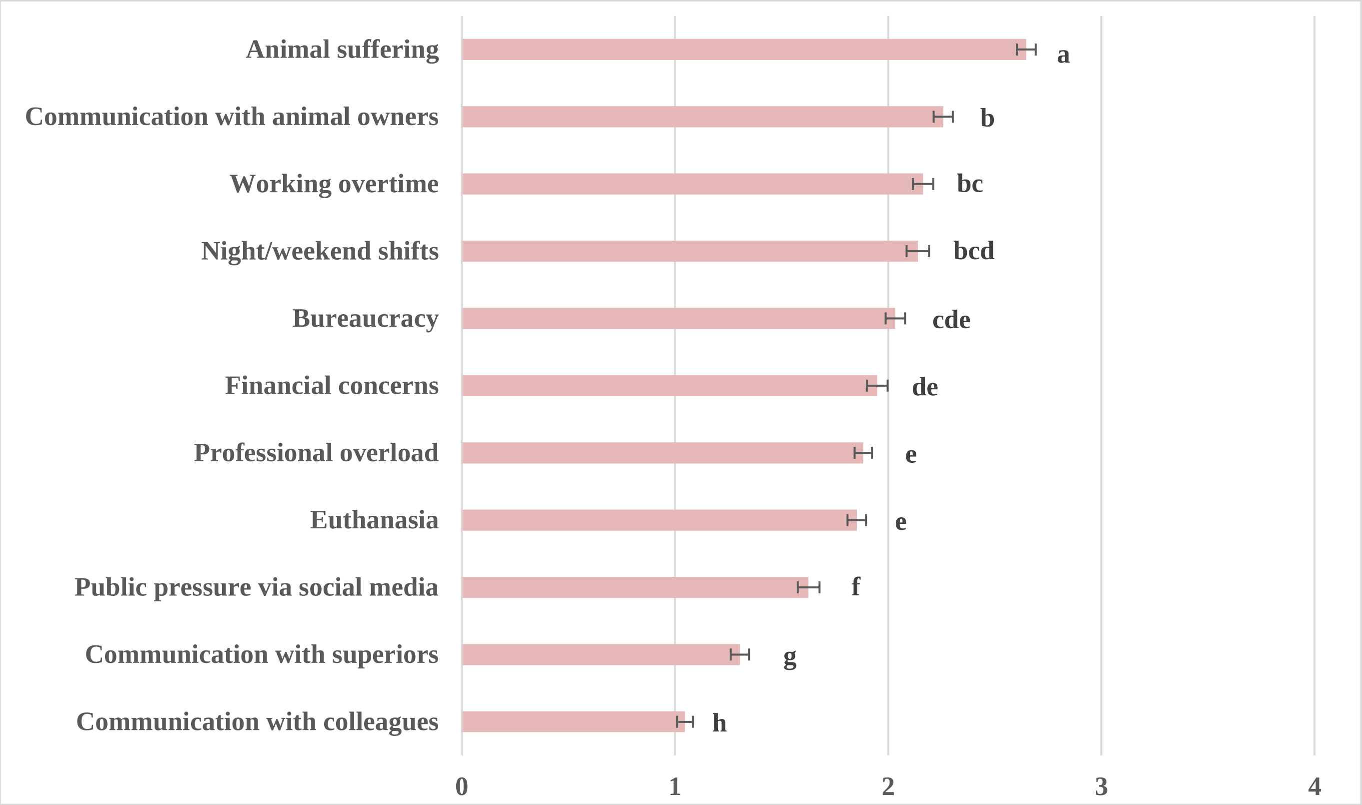

Supplementary Figure 3 Estimated/experienced veterinary work-related stressors in female veterinary students and veterinarians. The eleven pre-defined work-related stressors were rated on a 5-point scale from 0 ‘not at all or not applicable’ to 4 ‘very strongly’. Different letters (a,b,c,d,e,f,g,h) indicate statistically significant differences between stressors. Stressors with different letters are significantly different from each other (*P* < 0.05 after Bonferroni-correction).

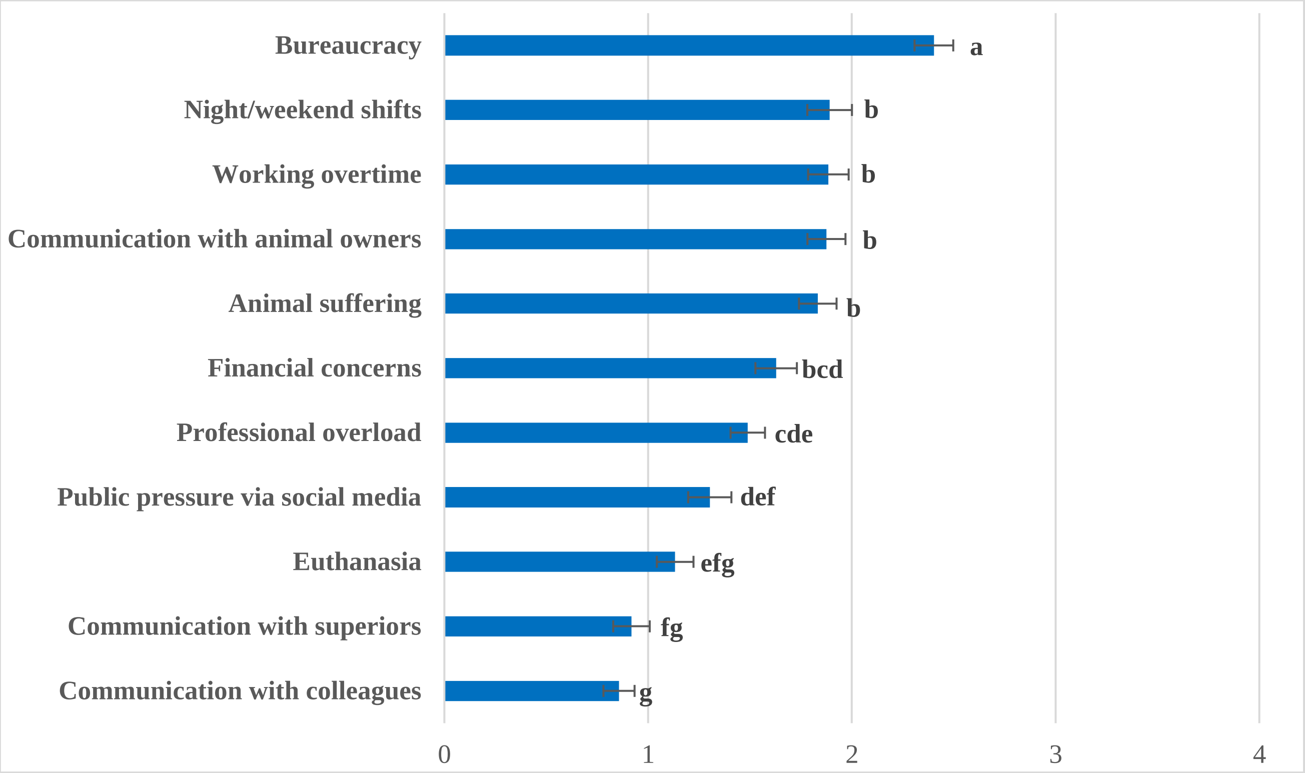

Supplementary Figure 4 Estimated/experienced veterinary work-related stressors in male veterinary students and veterinarians. The eleven pre-defined work-related stressors were rated on a 5-point scale from 0 ‘not at all or not applicable’ to 4 ‘very strongly’. Different letters (a,b,c,d,e,f,g) indicate statistically significant differences between stressors. Stressors with different letters are significantly different from each other ($P < 0.05$ after Bonferroni-correction).

Suppl. Table 1 Work-related stressors in veterinarians related to employment status

Stressor	Self-employment	N	Mean	SD	p-Value
**Communication with animal owners**	**no**	179	2.12	1.264	0.502
	**yes**	261	2.03	1.272	
**Communication with colleagues**	**no**	179	0.93	0.924	0.663
	**yes**	261	0.89	1.046	
**Communication with superiors**	**no**	**179**	**1.50**	**1.396**	**<0.001**
	**yes**	**261**	**0.81**	**0.874**	
**Night/weekend shifts**	**no**	179	2.07	1.386	0.125
	**yes**	261	1.87	1.341	
**Working overtime**	**no**	179	1.93	1.234	0.445
	**yes**	261	1.84	1.200	
**Euthanasia**	**no**	179	1.42	1.070	0.922
	**yes**	261	1.41	1.217	
**Animal suffering**	no	179	2.40	1.269	0.027
	yes	261	2.12	1.268	
**Bureaucracy**	**no**	**179**	**1.87**	**1.265**	**<0.001**
	**yes**	**261**	**2.87**	**1.139**	
**Professional overload**	**no**	**179**	**1.63**	**1.146**	**<0.001**
	**yes**	**261**	**1.14**	**1.029**	
**Financial concerns**	**no**	179	1.60	1.376	0.461
	**yes**	261	1.51	1.352	
**Public pressure via social media**	**no**	179	1.51	1.342	0.514
	**yes**	261	1.42	1.392	

Note: Experienced work-related stressors were rated on a 5-point scale from 0 ‘not at all or not applicable’ to 4 ‘very strongly’.

Suppl. Table 2 Work-related stressors in veterinarians related to professional field

Stressor	Curative practice	N	Mean	SD	p-Value
**Communication with animal owners**	**no**	53	1.81	1.442	0.165
	**yes**	387	2.10	1.240	
**Communication with colleagues**	**no**	53	0.83	0.826	0.575
	**yes**	387	0.91	1.019	
**Communication with superiors**	**no**	53	1.45	1.462	0.052
	**yes**	387	1.04	1.111	
**Night/weekend shifts**	**no**	**53**	**1.34**	**1.159**	**<0.001**
	**yes**	**387**	**2.04**	**1.367**	
**Working overtime**	no	53	1.53	1.154	0.024
	yes	387	1.93	1.215	
**Euthanasia**	**no**	53	1.36	1.162	0.690
	**yes**	387	1.43	1.159	
**Animal suffering**	**no**	53	2.25	1.426	0.951
	**yes**	387	2.23	1.254	
**Bureaucracy**	**no**	53	2.00	1.286	0.005
	**yes**	387	2.52	1.278	
**Professional overload**	**no**	53	0.98	1.009	0.008
	**yes**	387	1.39	1.108	
**Financial concerns**	**no**	53	1.23	1.423	0.069
	**yes**	387	1.59	1.348	
**Public pressure via social media**	**no**	53	1.38	1.417	0.653
	**yes**	387	1.47	1.366	

Note: Experienced work-related stressors were rated on a 5-point scale from 0 ‘not at all or not applicable’ to 4 ‘very strongly’.

Suppl. Table 3 Work-related stressors in veterinarians related to working with ruminants

Stressor	Working with ruminants	N	Mean	SD	p-Value
**Communication with animal owners**	**no**	287	2.19	1.232	0.006
	**yes**	153	1.84	1.308	
**Communication with colleagues**	**no**	287	0.93	0.999	0.480
	**yes**	153	0.86	0.996	
**Communication with superiors**	**no**	287	1.09	1.112	0.963
	**yes**	153	1.08	1.262	
**Night/weekend shifts**	**no**	287	1.87	1.363	0.074
	**yes**	153	2.11	1.350	
**Working overtime**	**no**	287	1.87	1.198	0.841
	**yes**	153	1.90	1.247	
**Euthanasia**	**no**	287	1.52	1.155	0.009
	**yes**	153	1.22	1.143	
**Animal suffering**	**no**	287	2.25	1.261	0.648
	**yes**	153	2.20	1.303	
**Bureaucracy**	**no**	**287**	**2.28**	**1.276**	**<0.001**
	**yes**	**153**	**2.80**	**1.246**	
**Professional overload**	**no**	287	1.38	1.086	0.327
	**yes**	153	1.27	1.136	
**Financial concerns**	**no**	**287**	**1.68**	**1.367**	**0.004**
	**yes**	**153**	**1.29**	**1.316**	
**Public pressure via social media**	**no**	287	1.50	1.371	0.386
	**yes**	153	1.38	1.372	

Note: Experienced work-related stressors were rated on a 5-point scale from 0 ‘not at all or not applicable’ to 4 ‘very strongly’.

Suppl. Table 4 Work-related stressors in veterinarians related to working with pigs

Stressor	Working with pigs	N	Mean	SD	p-Value
**Communication with animal owners**	**no**	353	2.09	1.249	0.455
	**yes**	87	1.98	1.347	
**Communication with colleagues**	**no**	353	0.91	1.009	0.675
	**yes**	87	0.86	0.954	
**Communication with superiors**	**no**	353	1.05	1.136	0.172
	**yes**	87	1.25	1.269	
**Night/weekend shifts**	**no**	353	1.94	1.376	0.782
	**yes**	87	1.99	1.307	
**Working overtime**	**no**	353	1.87	1.220	0.732
	**yes**	87	1.92	1.193	
**Euthanasia**	**no**	353	1.43	1.151	0.651
	**yes**	87	1.37	1.192	
**Animal suffering**	**no**	353	2.22	1.267	0.664
	**yes**	87	2.29	1.311	
**Bureaucracy**	**no**	**353**	**2.36**	**1.274**	**0.001**
	**yes**	**87**	**2.87**	**1.274**	
**Professional overload**	**no**	353	1.37	1.088	0.177
	**yes**	87	1.20	1.160	
**Financial concerns**	**no**	353	1.62	1.366	0.020
	**yes**	87	1.24	1.303	
**Public pressure via social media**	**no**	353	1.47	1.375	0.744
	**yes**	87	1.41	1.360	

Note: Experienced work-related stressors were rated on a 5-point scale from 0 ‘not at all or not applicable’ to 4 ‘very strongly’.

Suppl. Table 5 Work-related stressors in veterinarians related to working with poultry

Stressor	Working with poultry	N	Mean	SD	p-Value
**Communication with animal owners**	**no**	395	2.08	1.255	0.452
	**yes**	45	1.93	1.388	
**Communication with colleagues**	**no**	395	0.91	1.016	0.682
	**yes**	45	0.84	0.824	
**Communication with superiors**	**no**	395	1.07	1.152	0.417
	**yes**	45	1.22	1.277	
**Night/weekend shifts**	**no**	395	2.00	1.366	0.039
	**yes**	45	1.56	1.271	
**Working overtime**	**no**	395	1.91	1.220	0.133
	**yes**	45	1.62	1.134	
**Euthanasia**	**no**	395	1.41	1.157	0.666
	**yes**	45	1.49	1.180	
**Animal suffering**	**no**	395	2.21	1.261	0.296
	**yes**	45	2.42	1.390	
**Bureaucracy**	**no**	395	2.46	1.282	0.785
	**yes**	45	2.51	1.359	
**Professional overload**	**no**	395	1.37	1.108	0.069
	**yes**	45	1.07	1.031	
**Financial concerns**	**no**	**395**	**1.61**	**1.371**	**0.003**
	**yes**	**45**	**1.02**	**1.158**	
**Public pressure via social media**	**no**	395	1.44	1.364	0.533
	**yes**	45	1.58	1.438	

Note: Experienced work-related stressors were rated on a 5-point scale from 0 ‘not at all or not applicable’ to 4 ‘very strongly’.

Suppl. Table 6 Work-related stressors in veterinarians related to working with pets

Stressor	Working with pets	N	Mean	SD	p-Value
**Communication with animal owners**	**no**	**103**	**1.70**	**1.297**	**0.001**
	**yes**	**337**	**2.18**	**1.239**	
**Communication with colleagues**	**no**	103	0.93	1.050	0.730
	**yes**	337	0.89	0.982	
**Communication with superiors**	**no**	103	1.02	1.180	0.491
	**yes**	337	1.11	1.161	
**Night/weekend shifts**	**no**	103	1.95	1.382	0.994
	**yes**	337	1.95	1.358	
**Working overtime**	**no**	103	1.86	1.321	0.883
	**yes**	337	1.88	1.181	
**Euthanasia**	**no**	103	1.28	1.150	0.172
	**yes**	337	1.46	1.159	
**Animal suffering**	**no**	103	2.11	1.305	0.247
	**yes**	337	2.27	1.264	
**Bureaucracy**	**no**	103	2.50	1.298	0.696
	**yes**	337	2.45	1.288	
**Professional overload**	**no**	**103**	**0.99**	**0.934**	**<0.001**
	**yes**	**337**	**1.45**	**1.130**	
**Financial concerns**	**no**	**103**	**1.15**	**1.256**	**<0.001**
	**yes**	**337**	**1.67**	**1.370**	
**Public pressure via social media**	**no**	**103**	**1.13**	**1.186**	**0.002**
	**yes**	**337**	**1.56**	**1.409**	

Note: Experienced work-related stressors were rated on a 5-point scale from 0 ‘not at all or not applicable’ to 4 ‘very strongly’.

Suppl. Table 7 Work-related stressors in veterinarians related to working with horses

Stressor	Working with horses	N	Mean	SD	p-Value
**Communication with animal owners**	**no**	276	2.04	1.267	0.597
	**yes**	164	2.11	1.273	
**Communication with colleagues**	**no**	276	0.89	0.983	0.841
	**yes**	164	0.91	1.024	
**Communication with superiors**	**no**	276	1.06	1.130	0.474
	**yes**	164	1.14	1.223	
**Night/weekend shifts**	**no**	**276**	**1.81**	**1.389**	**0.004**
	**yes**	**164**	**2.20**	**1.282**	
**Working overtime**	**no**	276	1.79	1.226	0.054
	**yes**	164	2.02	1.182	
**Euthanasia**	**no**	276	1.37	1.129	0.254
	**yes**	164	1.50	1.206	
**Animal suffering**	**no**	276	2.17	1.214	0.173
	**yes**	164	2.34	1.368	
**Bureaucracy**	**no**	**276**	**2.30**	**1.311**	**<0.001**
	**yes**	**164**	**2.73**	**1.210**	
**Professional overload**	**no**	276	1.41	1.113	0.081
	**yes**	164	1.22	1.080	
**Financial concerns**	**no**	276	1.53	1.352	0.798
	**yes**	164	1.57	1.380	
**Public pressure via social media**	**no**	276	1.47	1.381	0.834
	**yes**	164	1.44	1.358	

Note: Experienced work-related stressors were rated on a 5-point scale from 0 ‘not at all or not applicable’ to 4 ‘very strongly’.

Suppl. Table 8 Work-related stressors in veterinarians related to working with exotic animals

Stressor	Working with exotic animals	N	Mean	SD	p-Value
**Communication with animal owners**	**no**	374	2.05	1.251	0.494
	**yes**	66	2.17	1.365	
**Communication with colleagues**	**no**	374	0.91	1.012	0.836
	**yes**	66	0.88	0.920	
**Communication with superiors**	**no**	374	1.05	1.126	0.129
	**yes**	66	1.32	1.349	
**Night/weekend shifts**	no	374	2.02	1.371	0.008
	yes	66	1.55	1.243	
**Working overtime**	**no**	374	1.90	1.224	0.506
	**yes**	66	1.79	1.157	
**Euthanasia**	**no**	374	1.43	1.166	0.765
	**yes**	66	1.38	1.120	
**Animal suffering**	**no**	374	2.22	1.248	0.735
	**yes**	66	2.29	1.423	
**Bureaucracy**	**no**	374	2.43	1.287	0.232
	**yes**	66	2.64	1.297	
**Professional overload**	**no**	374	1.36	1.116	0.313
	**yes**	66	1.21	1.031	
**Financial concerns**	**no**	374	1.55	1.361	1.000
	**yes**	66	1.55	1.372	
**Public pressure via social media**	**no**	374	1.44	1.348	0.445
	**yes**	66	1.58	1.499	

Note: Experienced work-related stressors were rated on a 5-point scale from 0 ‘not at all or not applicable’ to 4 ‘very strongly’.

Suppl. Table 9 Pearson correlation analyses investigating associations of the expected burden of work-related stressors and indicators of mental health in Austrian veterinary students (N = 430)

Stressor	Depression (PHQ-9)	Anxiety (GAD-7)	Insomnia (ISI-2)	Well-being (WHO-5)	Stress (PSS-4)
Communication with animal owners	.151	.124	.154	-.178*	0.092
Communication with colleagues	.209*	.185*	.148	-.225*	.243*
Communication with superiors	.286*	.250*	.189*	-.284*	.289*
Night/weekend shifts	.159*	.095	.099	-.160*	0.086
Working overtime	.254*	.181*	.162*	-.249*	.195*
Euthanasia	0.093	0.079	.103	-0.078	.121
Animal suffering	.200*	.208*	.118	-.152	.175*
Bureaucracy	0.090	0.073	0.032	-0.062	.115
Professional overload	.280*	.248*	.124	-.200*	.276*
Financial concerns	.292*	.243*	.156	-.235*	.240*
Public pressure via social media	.233*	.250*	.178*	-.119	.213*

Notes: *. The correlation is significant after correcting for multiple testing *(P* < 0.05/55 correlation analyses). Work-related stressors were rated on a 5-point Likert scale ranging from 0 ‘not at all’ to 4 ‘very strongly’.
